# Supplementary material for: A Review of Lipidomics of Cardiovascular Disease Highlights the Importance of Isolating Lipoproteins
Source: Metabolites. 2020 Apr 23;10(4):163. doi: 10.3390/metabo10040163 (PMC7240942; doi:10.3390/metabo10040163)
Supplement: Supplementary file 1 [file metabolites-10-00163-s001.pdf]

Table S1. A summary of lipidomic-associated studies with cardiovascular disease.

Study name, number of participants, country, publication year	Study design	Platform	Lipids positively associated with the outcome	Lipids inversely associated with the outcome	Lipid biomarkersadj usted for	Other covariates adjusted for
Malmo Diet and Cancer (MDC) study,[17]Sweden,2013,n=427	Nested case control study	Top-down shotgun lipidomics 85 lipids	Without correcting for multiple testing, SM38:2 was positively associated with risk of CVD.None identified after correcting for multiple testing.	Without correcting for multiple testing, LPC16:0, LPC20:4, TAG48:1, TAG48:2, TAG48:3, TAG50:3 and TAG50:4, were inversely associated with risk of CVD.None identified after correcting for multiple testing.	HDL-c and LDL-c	Sex, age, BMI, type 2 diabetes, anti-hypertension treatment, smoking, and SBP
Bruneck Study,[18]Italy,2014, n=1400	Cohort study	Top-down shotgun lipidomics135 lipids	Lipid species with the strongest positive association with risk of CVD were TAGs and CEs with a low carbon number and double-bond content.28 lipids were identified, namely, TAG(50:1), TAG(50:2), TAG(50:3), TAG(52:2), TAG(52:3), TAG(52:5), TAG(54:2), TAG(56:1), TAG(56:5), TAG(56:6), PE(34:1), PE(34:2), PE(36:2), PE(36:4), PE(36:5), PE(38:3), PE(38:4), PE(38:5), PE(O-38:5), SM(34:2), SM(42:2), PC(32:1), PC(38:2), PC(38:3), CE(14:0), CE(16:0), CE(16:1), CE(22:2)	None.	Statin medication	Age and sex
TwinGene, ULSAM, and PIVUS,[25]Sweden,2014,n=3668	Nested case control study	LC-MS 21 lipids	MAG 18:2 was positively associated with risk of CVD.	LPC 18:1, LPC 18:2, and SM 28:1 were inversely associated with risk of CVD.	LDL-c, HDL-c, TAG	Sex, systolic blood pressure, body mass index, current smoker, antihypertensive treatment, and diabetes at baseline.
LURIC study,[21]Germany, 2014,n=3316	Cohort studyThe majority had a history of CHD.	LC-MS178 lipids	Without correcting for multiple testing, 30 lipids were positively associated with risks of recurrent CHD and mortality, including PC(32:0), PC(38:0), PC(30:1), PC(34:1), PC(36:1), PC(O-32:0), PC(O-34:0), PC(O-32:1), PC(O-34:1), PC(O-38:5), PE(30:1), PE(32:1), PE(34:1), PE(36:1), PE(40:1), PE(34:2), PE(36:2), PE(38:2), PE(34:3), PE(36:3), PE(36:4), PE(O-32:0), PE(O-36:2), SM(16:0), SM(16:1), SM(24:1), SM(24:2), Cer(d18:1/16:0), Cer(d18:1/18:0), and Cer(d18:1/24:1)	Without correcting for multiple testing, 18 lipids were inversely associated with risks of recurrent CHD and mortality, including PC(32:2), PC(36:4), PC(36:5), PC(40:6), PC(40:7), PC(38:7), PC(38:6), PC(38:5), PC(38:4), PC(38:3), LPC(16:0), LPC(18:0),PE(36:6),PE(O-38:7), SM(23:0), SM(24:0), Cer(d18:1/23:0), and Cer(d18:1/24:0)	HDL-c and LDL-c	Age, gender, smoking, diabetes and hypertension.
ATHEROREMO-IVUS,[26,27]Netherland,2015 and 2018,n=581	Cohort studyAll participants had a history of CHD.	LC-MS Lipids identified in LURIC study, including CE 14:0, CE 18:3, CE 20:4, CE 20:5, and CE 22:5,	Cer (d18:1/16:0) and Cer(d18:1/24:1)/Cer(d18:1/24:0) ratio were positively associated with major adverse cardiac events including recurrent CHD and all-cause mortality.	None.	Statin use, LDL-c	Gender, age, hypertension, hypercholesterolemia, and diabetes.

		Cer(d18:1/16:0), Cer(d18:1/20:0), Cer(d18:1/24:0), Cer(d18:1/24:1),and lactosylceramide (LacCer, d18:1/18:0).				
FINRISK, SABRE, and BWHHS,[19]Finland and U.K.,2015,n=13441	Cohort study	NMR Total lipid within 14 lipoprotein subclasses; triglycerides, phospholipids, sphingomyelin, and fatty acyl chain in total plasma	Before adjusting for standard lipids, the total lipid concentrations within VLDL, IDL, and LDL were positively associated with risk of CVD.After further adjusting for routine lipids, only MUFA was positively associated with risk of CVD.	Before adjusting for standard lipids, total lipid concentrations and cholesterols within large HDL particles were inversely associated with risk of CVD.After further adjusting for routine lipids, PUFA, omega-6 fatty acids, and DHA were inversely associated with risk of CVD.	Total and HDL-cholesterol	Age, sex, blood pressure, smoking, diabetes mellitus, geographical region, and cardiovascular medication.
FINRISK 2002 Cohort,[28] Finland,2016,n=8101	Cohort study	LC-MS Four ceramides including Cer(d18:1/16:0),Cer(d18:1/18:0), Cer(d18:1/24:0), and Cer(d18:1/24:1)	Cer(d18:1/16:0), Cer(d18:1/18:0), and Cer(d18:1/24:1) were positively associated with risk of CVD.	None.	Total and HDL-cholesterol	Blood pressure (adjusted +15 mm Hg for antihypertensive medication), diabetes mellitus, and smoking.
Corogene study,SPUM-ACS, and BECAC,[29]Northern European countries,2016,n=3377	Cohort study.The majority had a history of CHD.	LC-MS Four ceramides including Cer(d18:1/16:0),Cer(d18:1/18:0), Cer(d18:1/24:0), and Cer(d18:1/24:1)	Cer(d18:1/16:0) and Cer(d18:1/16:0)/Cer(d18:1/24:0) ratio were positively associated with higher risk of CVD mortality.	Cer(d18:1/24:0) was inversely associated with risk of CVD mortality.	Total cholesterol, TAG, LDL-c, and HDL-c	Age, gender, smoking status, previous acute MI, diabetes,hypertension, and prior stroke.
ADVANCE trial,[22]Australia,2016,n=3779	Case-cohort study.All participants had a history of CVD.	LC-MS 310 lipids	17 ceramides, 1 SM, 7 PCs, 8 LPCs, and 3 CEs were positively associated with risks of recurrent CVD incidence and CVD mortality, namely, Cer(d18:1/24:1), HexCer (d18:1/16:0), HexCer (d18:1/18:0), HexCer (d18:1/20:0), HexCer (d18:1/22:0), HexCer (d18:1/24:0), HexCer (d18:1/24:1), Hex2Cer (d18:1/16:0), Hex2Cer (d18:1/18:0), Hex2Cer (d18:1/20:0), Hex2Cer (d18:1/22:0), Hex2Cer (d18:1/24:0), Hex2Cer (d18:1/24:1), Hex3Cer (d18:1/20:0), Hex3Cer (d18:1/22:0), Hex3Cer (d18:1/24:0), Hex3Cer (d18:1/24:1), SM (34:1), PC(O-32:0), PC(O-32:1), PC(O-34:1), PC(O-36:1), PC(O-36:2), PC(P-32:1), PC(P-34:1), LPC (20:1), LPC (O-18:0), LPC (O-18:1), LPC (O-22:0), LPC (O-22:1), LPC (O-24:0), LPC (O-24:1), LPC (O-24:2), CE (16:0), CE (20:1), and CE (24:1)	5 PCs and 1 TAG were inversely associated with risks of recurrent CVD incidence and CVD mortality, namely, PC (34:5), PC(35:4), PC (40:6), PC (P-36:5), PC (P-38:6),TAG (56:6)	HDL-c	Age, sex, body mass index, systolic blood pressure, glycohemoglobin, estimated glomerular filtration rate, diabetes duration, C-reactiveprotein, history of macrovascular disease, history of heart failure, use of antihypertensive medication, use of antiplatelet medication, and exercise.

PREDIMEDTrial,[30-32]Spain,2017 and 2018, n=980	Case-cohort study	LC-MS202 lipids	Cer (d18:1/16:0), Cer (d18:1/22:0), Cer (d18:1/24:0), and Cer (d18:1/24:1) were positively associated with risk of CVD.Lipid cluster that included DAG and MAG with stearic acyl chain was positively associated with risk of CVD.	Lipid cluster that includedhighly unsaturated PCs and CEs was inversely associated with risk of CVD.	History of dyslipidemia	Age, sex, bodymass index, family history of premature CHD, smoking status,histories of hypertension, and type 2 diabetes mellitus.
Women's Health Initiative,[23]U.S.,2017,n=1571	Nested case control study	LC-MS217 lipids	After adjusting for standard lipids, 5 lipids including C34:2 hydroxy-PC, C36:4 hydroxy-PC, 5-hydroxyeicosatetraenoic acid(HETE), 15-HETE, and 11-HETE were positively associated with risk of CVD.TAG and DAG were positively associated with risk of CVD, however, the association was eliminated after adjusting for standard lipids.	None.	Total and HDLcholesterol	Baseline age, race/ethnicity, hysterectomy status, enrollment window, aspirin use, statin use,antihyperglycemic use, antihypertensive use, smoking, systolic blood pressure, and diabetes mellitus
China Kadoorie Biobank,[20]Chinese ,2018,n=4662	Nested case control study	NMR Total lipid, triglycerides, and cholesterol within 14 lipoprotein subclasses; fatty acyl chain in total plasma	Total lipid concentrations of VLDL, IDL, LDL were positively associated with risk of CVD. Triglycerides within all lipoproteins, including most HDL particles, were positively associated with risk of CVD.	Total lipid concentration and cholesterol in HDL were inversely associated with risk of CVD.	None	Age, sex, fasting hours, region, smoking status, and educationalattainment.
LIPID study,[24]Australia, 2018,n=5991	Cohort studyAll participants had a history of CHD.	LC-MS342 lipids	17 lipids were positively associated with risk of recurrent CVD, namely, LPC(O-24:1), LPC(O-24:2), LPC(O-18:0), LPC(O-18:1), PC(O-36:1), PC(O-34:1), PC(O-32:0), Hex3Cer(d18:1/22:0), Hex3Cer(d18:1/24:0), Hex3Cer(d18:1/24:1)Hex2Cer(d18:1/24:1), HexCer(d18:1/24:1)Hex2Cer(d18:1/16:0), Hex2Cer(d18:1/18:0)HexCer(d18:1/16:0), HexCer(d18:1/18:0)HexCer(d18:1/20:0)	13 lipids were inversely associated with risk of recurrent CVD, namely, PC(P-38:6), PC(P-36:5),PC(40:8), PC(40:5), PC(40:6), PC(38:4), PC(38:5), PC(38:6),PC(38:7), PC(37:6), PC(35:4), PC(34:4), PC(34:5)	Total and HDL-cholesterol	Age, sex, current smoking, nature of prior acute coronary syndrome, revascularization, diabetes history, stroke history, history of hypertension, and randomized treatment allocation.

ULSAM: Uppsala Longitudinal Study of Adult Men; PIVUS: Prospective Investigation of the Vasculature in Uppsala Seniors; LURIC: LUdwigshafen RIsk and Cardiovascular Health; SABRE: Southall And Brent Revisited Study; BWHHS: The British Women's Heart & Health Study; SPUM-ACS: Inflammation and Acute Coronary Syndromes Study; BECAC: Bergen Coronary Angiography Cohort; ADVANCE trial: the Action in Diabetes and Vascular Disease: Preterax and Diamicron MR Controlled Evaluation (ADVANCE) trial; PREDIMED trial: The Prevención con Dieta Mediterránea (PREDIMED) trial; NMR: Nuclear magnetic resonance; LC-MS: Liquid chromatography–mass spectrometry; PC: Phosphatidylcholine; LPC: Lysophosphatidylcholine; SM: Sphingomyelin; TAG: Triglycerides; DAG: Diglycerides; MAG: Monoglycerides; PE: Phosphatidylethanolamine; CE: Cholesterol esters; VLDL: Very low-density lipoprotein; IDL: Intermediate-density lipoprotein; LDL: Low-density lipoprotein; HDL: High-density lipoprotein; HexCer: Hexoceramide; Hex2Cer: Dihexoceramide; Hex3Cer: Trihexoceramide.
